# Analgesic efficacy of erector spinae plane block versus transversus abdominis plane block for laparoscopic cholecystectomy: a systematic review and meta-analysis of randomized controlled trial

**DOI:** 10.3389/fmed.2024.1399253

**Published:** 2024-07-29

**Authors:** Dereje Zewdu, Temesgen Tantu, Shamil Eanga, Tamiru Tilahun

**Affiliations:** ^1^Department of Anesthesia, College of Medicine and Health Science, Wolkite University, Wolkite, Ethiopia; ^2^Department of Obstetrics and Gynecology, College of Medicine and Health Science, Wolkite University, Wolkite, Ethiopia

**Keywords:** erector spinae plane block, transversus abdominis plane block, laparoscopic cholecystectomy, postoperative analgesia, nerve block analgesia

## Abstract

**Background:**

Although laparoscopic cholecystectomy (LC) is a minimally invasive surgery, it can cause moderate to severe postoperative pain. Erector spinae plane (ESP) and transversus abdominis plane (TAP) blocks are considered effective means for pain management in such cases; however, there is inconclusive evidence regarding their analgesic efficacy. This meta-analysis aimed to compare the efficacy of ESP block and TAP block for pain control in LC.

**Methods:**

We systematically searched Medline, PubMed, Scopus, Embase, and Google Scholar until 26 January 2024. All randomized clinical trials compared the efficacy of erector spinae plane block (ESPB) and transversus abdominis plane block (TAPB) for postoperative pain relief after LC. The primary outcomes were pain scores at rest and on movement at 1, 2, 6, 12, and 24 h postoperatively. The secondary outcomes were total opioid consumption, first analgesia request time, and rates of postoperative nausea and vomiting. We analyzed all the data using RevMan 5.4.

**Results:**

A total of 8 RCTs, involving 542 patients (271 in the ESPB group and 271 in the TAPB group), were included in the analysis. The ESP block demonstrated statistically significant lower pain scores at rest and on movement than the TAP block at all-time points except at the 1st and 6th h on movement postoperatively; however, these differences were not considered clinically significant. Additionally, patients who received the ESP block required less morphine and had a longer time before requesting their first dose of analgesia. There were no significant differences in postoperative nausea and vomiting incidence between the two groups.

**Conclusion:**

In patients undergoing LC, there is moderate evidence to suggest that the ESP block is effective in reducing pain severity, morphine equivalent consumption, and the time before the first analgesia request when compared to the TAP block during the early postoperative period.

**Systematic review registration:** PROSPERO CRD42024505635, https://www.crd.york.ac.uk/prospero/display_record.php?ID=CRD42024505635.

## 1 Introduction

Laparoscopic cholecystectomy (LC) is a frequently performed abdominal surgery. Although LC is less invasive and results in less postoperative pain than open cholecystectomy, it is not a pain-free procedure (1).

The fact that postoperative pain after LC is clinically significant is due to multiple sources of pain: visceral pain from gallbladder resection and peritoneal CO2 insufflation, and somatic pain from skin incisions (2, 3). Given this, providing proper pain management following LC is crucial, as poorly managed pain can lead to a range of complications, including prolonged hospital stays, readmissions, chronic postoperative pain, and persistent opioid use (4–6). Therefore, it is crucial to prioritize effective and safe pain management to improve postoperative outcomes in such cases.

Postsurgical pain management has been a topic of interest and controversy for many years. Several modalities are implemented into clinical practice to control postsurgical pain after LC, including patient-controlled analgesia, systemic opioids, and incision site infiltrations using local anesthetics and adjuvants (7–11). Although these methods have a proven analgesic advantage, their clinical significance remains uncertain due to their adverse effects, such as nausea, vomiting, respiratory depression, and urinary retention (11, 12).

Ultrasound-guided truncal blocks, such as transversus abdominis plane (TAP) and erector spinae plane blocks (ESPB), reportedly reduce the severity of postoperative pain and are effective alternatives to decrease cumulative opioid consumption and manage perioperative pain using multimodal analgesia in such cases (13–15).

Currently, ongoing meta-analyses (16, 17) are comparing the effectiveness of a transversus abdominis plane (TAP) block and an erector spinae plane (ESP) block to a placebo. The results of these analyses have shown that both the TAP block and the ESP block provide superior analgesia and reduce postoperative opioid consumption following laparoscopic cholecystectomy (LC). However, recent randomized clinical trials (18–25) have presented conflicting results when comparing the postoperative analgesic efficacy of ESP and TAP blocks after LC. While some studies have demonstrated the superior pain-relieving effects of the ESP block, others have reported no significant difference between the two treatment groups. Additionally, a recent review (26) compared the analgesic efficacy of ESP and TAP blocks after abdominal surgeries. However, the review had a main limitation: it included a range of surgical procedures that could result in varied pain intensity and anatomical differences, leading to significant heterogeneity.

Nevertheless, no trials have comprehensively analyzed the available data to compare the pain relief effectiveness of ESP and TAP blocks following LC. Therefore, we conducted a meta-analysis to assess the efficacy of an ESP block compared to a TAP block in providing postoperative pain relief for patients undergoing laparoscopic cholecystectomy.

## 2 Methods

This systematic review and meta-analysis was registered in the International Prospective Register of Systematic Review with registration number CRD42024505635 and was performed according to the eligibility criteria of the Preferred Reporting Items for Systematic Reviews and Meta-Analyses (PRISMA) recommendations (27).

### 2.1 Search strategy

We systematically searched potentially relevant publications in the following electronic databases: MEDLINE, PubMed, Scopus, Embase, and Google Scholar from inception to 26 January 2024. We manually retrieved relevant studies using keywords and references from identified studies and limited our search to articles published in English; however, we did not limit the year of publication.

**Figure 1:**
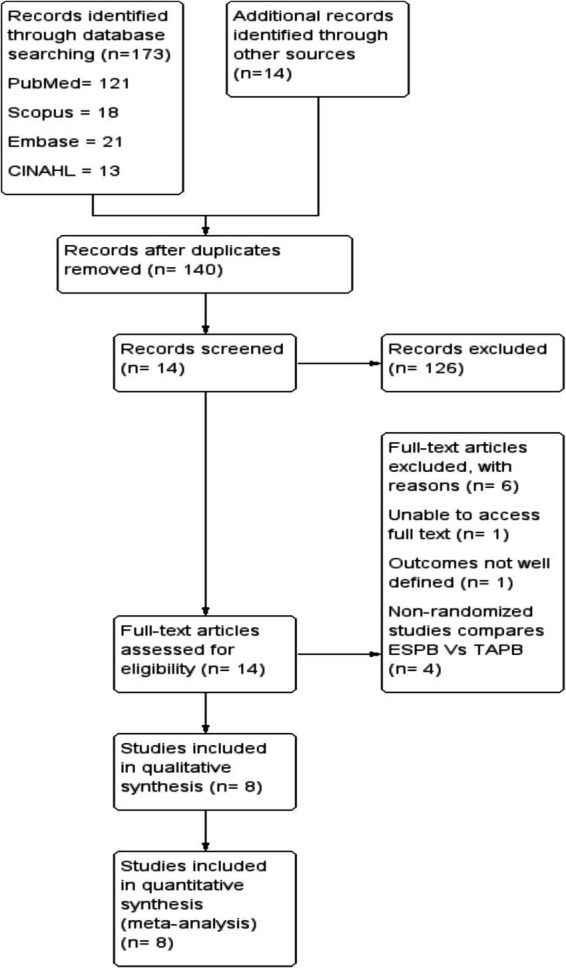
PRISMA flow diagram displaying the retrieved, included, and excluded studies.

The search strategy consisted of the following terms in combination with Boolean operators: ‘erector spinae plane block’, ‘transversus abdominis plane block’, and ‘laparoscopic cholecystectomy’. The search strategy for each database is attached as Supplementary File 1, and the retrieval process is illustrated in Figure 1.

### 2.2 Eligibility criteria

Studies that met the following criteria were considered eligible based on the PICOS criteria: (P) patients undergoing elective laparoscopic cholecystectomy (LC); (I) where the intervention group received ultrasound-guided ESP block; (C) the placebo or control group received TAP block; (O) postoperative pain scores, postoperative opioid consumption, first analgesia request time, and postoperative nausea and vomiting (PONV); and (S) randomized controlled trials.

The dosage, type, and volume of local anesthetics and the use of adjuvants in both groups did not affect the study’s eligibility. We excluded trials comparing ESP block versus TAP block for surgical procedures other than LC. We also excluded retrospective studies, non-randomized controlled trials, and studies focused on outcomes other than our interests.

### 2.3 Selection criteria

Two reviewers (SE and TT) independently reviewed the titles and abstracts of the potential publications. The full texts of the initially identified articles that potentially met the eligibility criteria were re-reviewed before the final decision. A third reviewer (DZ) made the final decision in cases of disagreement. Finally, the risk of bias was assessed for all included trials using the Review Manager (RevMan) software package, Version 5.4., Copenhagen.

**Figure 2:**
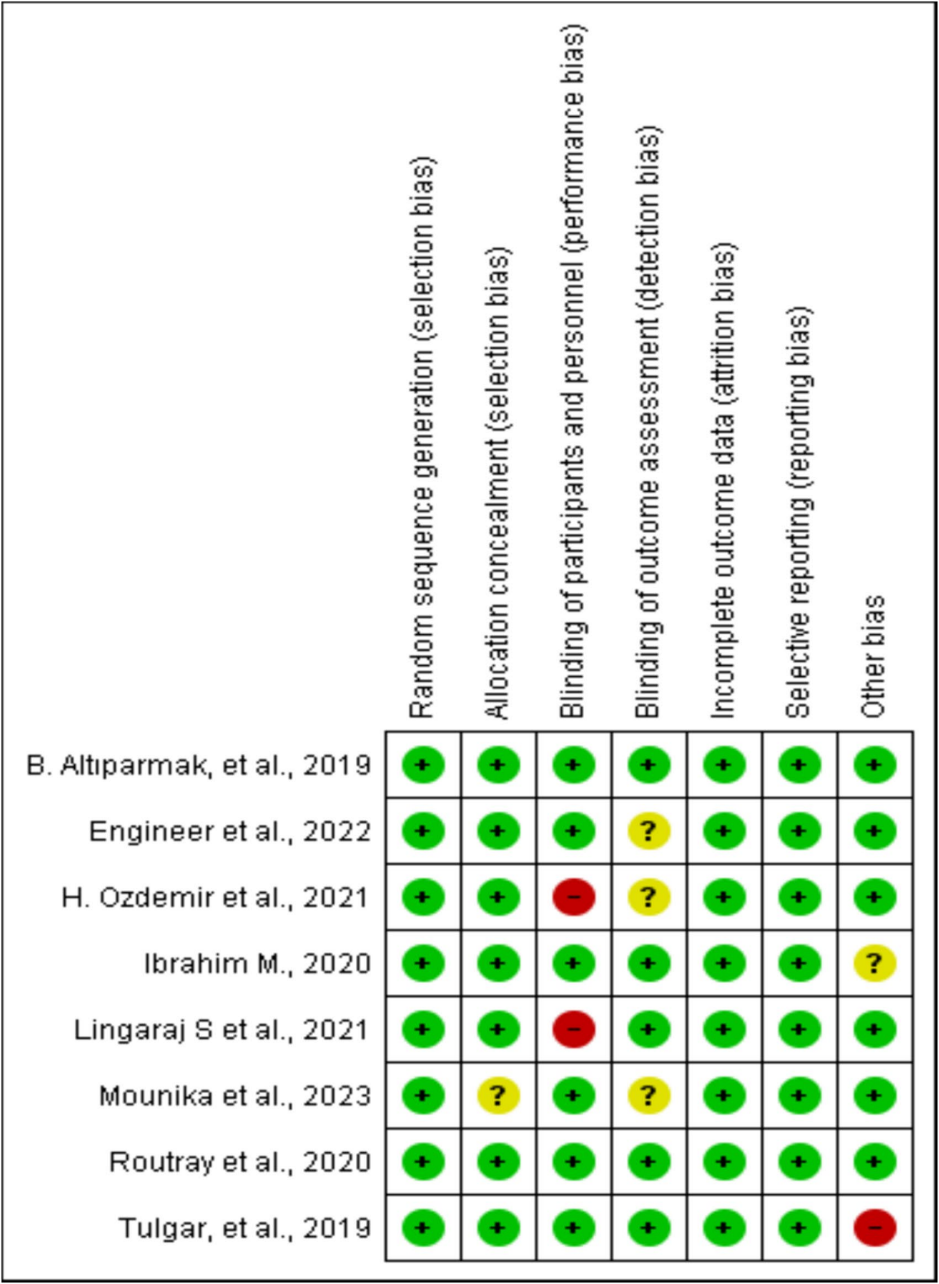
Risk of bias summary: review authors’ judgments about each risk of bias item for each included study.

This risk of bias tools included random sequence generation, concealment of treatment allocation, blinding throughout the study period, attrition, selective outcome reporting, and any other risk of bias, as presented in Figure 2.

### 2.4 Data extraction

Four reviewers (DZ, TT, TT, and SE) independently collected the relevant data from the included studies using a standardized data sheet. Extracted data included first author names, year of publication, study groups, local anesthetic dosage and types, adjuvants used, postoperative analgesia protocol, study outcomes, and other pertinent data from individual articles (Table 1).

**Table 1:** Patient’s characteristics of the included trials.

Author, year of publication	Patients characteristics	Sample size (ESPB/TAPB)	Local anesthetics (type, dose)	Block location	Block timing	Postoperative analgesia protocol	Outcomes
Altıparmak, et al. (21)	Patients aged 18–70 years, ASA I-II for elective LC	34/34	20 mL of 0.375% bupivacaine bilaterally for both blocks	ESPB at T7 Subcostal TAP block	Before incision	IV PCA of tramadol	Tramadol consumption, NRS pain score
Sahu et al. (19)	Patients aged 18–70 years, ASA I-II for elective LC	30/30	20 mL of 0.2% ropivacaine and 4 mg dexamethasone bilaterally for both blocks	ESPB at T7/T8 Subcostal TAP block	Awake states	iv Paracetamol iv Tramadol	Tramadol consumption, VAS pain score
Mounika et al. (20)	Patients aged 18–70 years, ASA I-II for elective LC	69/69	20 mL of 0.2% ropivacaine and 4 mg dexamethasone bilaterally for both blocks	ESPB at T7, Subcostal TAP block	Before incision	iv Paracetamol iv tramadol	Tramadol consumption, VAS pain score
Routray et al. (24)	Patients aged 18–65 years, ASA I-II for elective LC	35/35	20 mL of 0.375% bupivacaine bilaterally for both blocks	ESPB at T9, Subcostal TAP block	Before incision	iv Paracetamol	Paracetamol consumption, Analgesia request time, NRS pain score
Engineer et al. (18)	Patients aged 18–75 years, ASA I-III for elective LC	30/30	20 mL (10 mL of 0.375% bupivacaine and 10 mL of 1.5% lignoadrenaline) bilaterally for both blocks	ESPB at T9, Subcostal TAP block	Before incision	iv tramadol, im diclofenac	First analgesic request time, NRS pain score
Tulgar et al. (23)	Patients aged 18–65 years, ASA I-II for elective LC	20/20	20 mL (10 mL of bupivacaine 0.5%, 5 mL of lidocaine 2% and 5 mL normal saline) bilaterally for both blocks	ESPB at T9, Subcostal TAP block	Before incision	iv Paracetamol iv PCA of Tramadol, iv Fentanyl	Tramadol consumption, NRS pain score
Ozdemir et al. (22)	Patients aged 18–64 years, ASA I-III for elective LC	32/32	10 mL of 0.25% bupivacaine and 10 mL of 2% prilocaine bilaterally for both blocks	ESPB at T7, Subcostal TAP block	Awake states	iv Paracetamol iv Meperidine, iv PCA of fentanyl	NRS pain score Time to first analgesic request and fentanyl consumption.
Ibrahim (25)	Patients aged 20–60 years, ASA I-II for elective LC	21/21	20 mL of 0.25% bupivacaine bilaterally for both blocks	ESPB at T8, Subcostal TAP block	Before incision	iv Paracetamol iv PCA of Morphine	Morphine consumption, VAS score and first analgesic dose

We used standardized conversion equations to calculate the mean and standard deviation of the data presented as a median with an interquartile range or range (28). In the case of data displayed in a graphical format, we used plot digitization software (Plot Digitizer, 2.1, Free Software Foundation, Boston, MA, United States) to extract the numeric data.

### 2.5 Study outcomes

The primary outcome was the severity of pain scores at rest and during movement or coughing at 1, 2, 6, 12, and 24 h after surgery. Pain scores reported as visual, verbal, or numeric rating scale scores were converted to a standardized 0 to 10 analog scale for the quantitative evaluations. The secondary outcomes were the postoperative total consumption of morphine equivalents, first analgesia request time, and PONV at 24 h following LC. All types of opioids used for postoperative analgesia were converted to morphine equivalents (mg) using the British National Formulary standardized conversion tables (29).

For the primary outcomes of this review (i.e., the difference in pain score AUC from 1 to 24 h), we conducted a sensitivity analysis as planned. This involved sequentially excluding data from trials that were (1) published in non-indexed journals, (2) available only as abstracts, and (3) non-randomized clinical trials.

### 2.6 Statistical analysis

One author (SE) entered data into Review Manager 5.4.1 (Cochrane Library, Oxford, United Kingdom), and another author (DZ) checked it for statistical analysis. Continuous data, such as the severity of pain scores at different time points, total tramadol consumption, and first analgesia request time at 24 h after LC, were analyzed using mean difference (MD) or standard mean difference (SMD) with a 95% confidence interval (CI). On the other hand, dichotomous outcomes were analyzed using relative risk (RR) with a 95% confidence interval (CI). We performed a statistical heterogeneity analysis using Higgins’s I2 test. We used a random-effects model for the meta-analysis, regardless of the I2 results. A *p*-value of <0.05 was considered statistically significant for all tests. Whenever necessary, we conducted a sensitivity analysis to identify the sources of heterogeneity.

## 3 Results

### 3.1 Search results

We identified a total of 187 studies through electronic databases and manual searches. After removing duplicates, we screened the title and abstract of each article. Of these, 126 articles were excluded from the meta-analysis because they did not meet the inclusion criteria during title and abstract screening. Finally, 8 randomized controlled trials published between 2019 and 2023 comprising 542 patients met the inclusion criteria (Figure 1).

### 3.2 Risk of bias assessment

All the included studies were RCTs. We described trial characteristics and relevant details about the included articles (Table 1). We used RevMan software’s risk of bias tool for systematic reviews of interventions to evaluate the included trials (Figure 2). Clear eligibility criteria for each included trial and a detailed randomization step were summarized. Patients were blinded to the type of block they received, except in two trials, in which, ESP and TAP blocks were performed in an awake state (19, 21). In addition, there was an unknown risk of blinding of outcome assessment (18, 20, 22) and allocation concealment (20).

### 3.3 Study characteristics

The sample sizes in the included RCTs ranged from 40 to 138. All studies compared the efficacy of ESPB and TAPB for postoperative analgesia following LC. The intervention groups received ESPB, whereas the control groups received TAPB. All the included trials used standardized anesthesia induction protocols. Propofol was used for general anesthesia induction along with fentanyl (18, 21–25) and nalbuphine (19, 20). Three studies (21–23) used opioids with inhalational agents as a part of anesthesia maintenance, and the remainder used inhalational agents alone.

All patients, except in two trials (19, 22), received ESPB or TAPB under general anesthesia before the surgical incision. Dosages, types, drug mixtures, and adjuvants used with local anesthetics varied between studies; however, the same dose and type of drugs were administered for both blocks in all studies. All trials used a postoperative analgesia protocol for pain management; however, four studies (21–23, 25) used patient-controlled analgesia (PCA).

### 3.4 Outcomes in the meta-analysis

#### 3.4.1 Postoperative pain scores at rest and on movement during 24 h postoperatively

The primary outcome was the pain score at different time points (1, 2, 6, 12, and 24 h) postoperatively at rest and on active movement following LC.

##### 3.4.1.1 Postoperative pain scores at rest

**Figure 3:**
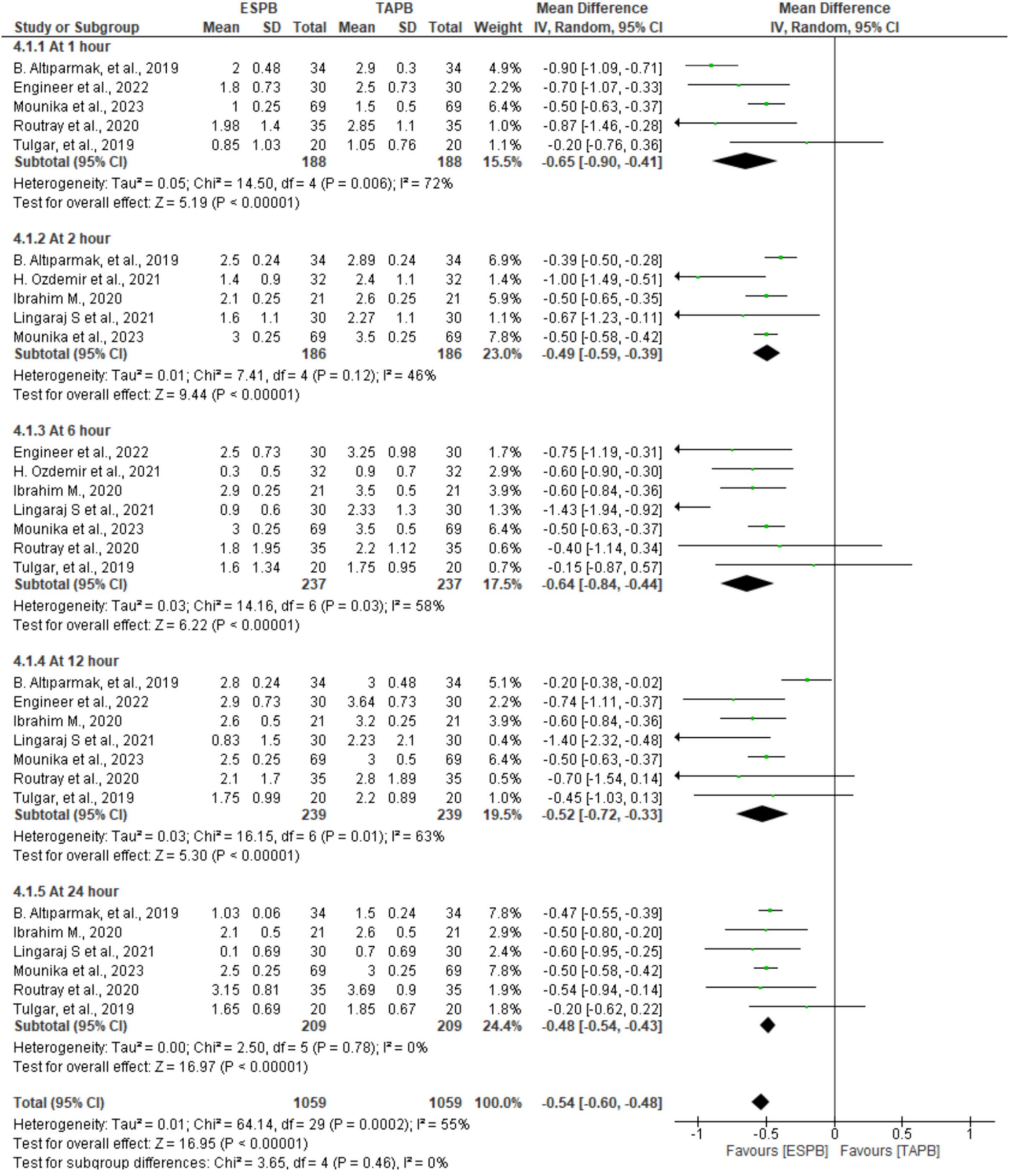
Postoperative pain scores at different time points at rest after LC.

In seven trials comprising patients between 372 and 478, the authors reported pain scores at different time points following LC. There was significant heterogeneity across the specified time points (I2 = 55%, *p* < 0.001); therefore, we used a random-effects model. The pooled results demonstrated that there was a statistically significant difference in favor of the ESPB group over the TAPB group in terms of postoperative pain score at 1 (MD = −0.65, 95% CI: −0.90 to −0.41; *p* < 0.001; I2 = 72%), 2 (MD = −0.49, 95% CI: −0.59 to −0.39; *p* < 0.001; I2 = 46%), 6 (MD = −0.64, 95% CI: −0.84 to −0.44; *p* < 0.001; I2 = 58%), 12 (MD = −0.52, 95% CI: −0.72 to −0.33; *p* < 0.001; I2 = 63%), and 24 h (MD = −0.48, 95% CI: −0.54 to −0.43; *p* < 0.001; I2 = 0%), as shown in Figure 3.

##### 3.4.1.2 Postoperative pain scores on movement

**Figure 4:**
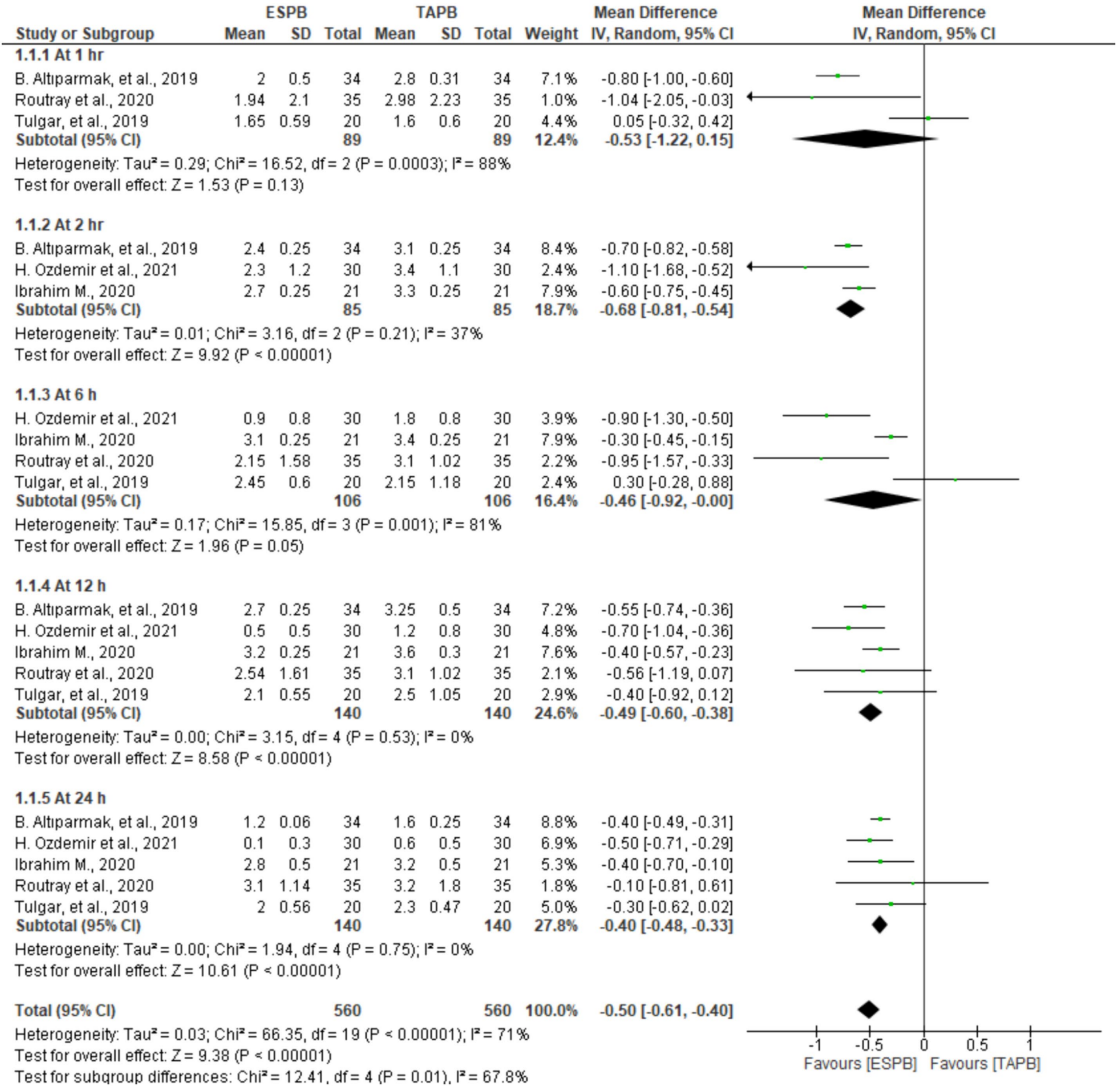
Postoperative pain scores at different time points on movement after LC.

Patients who underwent LC reported their postoperative pain scores at 1, 2, 6, 12, and 24 h after movement in five trials involving 128 to 280 patients. Significant heterogeneity was found between the studies at the predetermined time points (I2 = 71%, *p* < 0.001); therefore, a random-effects model was computed. The results of the meta-analysis revealed that the ESPB had significantly lower postoperative pain scores on movement at 2 (MD = −0.68, 95% CI: −0.81 to −0.54; *p* < 0.001; I2 = 37%), 12 (MD = −0.49, 95% CI: −0.60 to −0.38; *p* < 0.001; I2 = 0%), and 24 h (MD = −0.40, 95% CI: −0.48 to −0.33; *p* < 0.001; I2 = 0%) than the TAPB groups, as displayed in Figure 4. However, there were no significant differences between these two blocks at 1 (MD = −0.53, 95% CI: −1.22 to 0.15; *p* = 0.13; I2 = 88%) and 6 h (MD = −0.46, 95% CI: −0.92 to −0.00; *p* = 0.05; I2 = 81%).

#### 3.4.2 Intravenous morphine equivalent consumption at 24 h postoperatively

**Figure 5:**
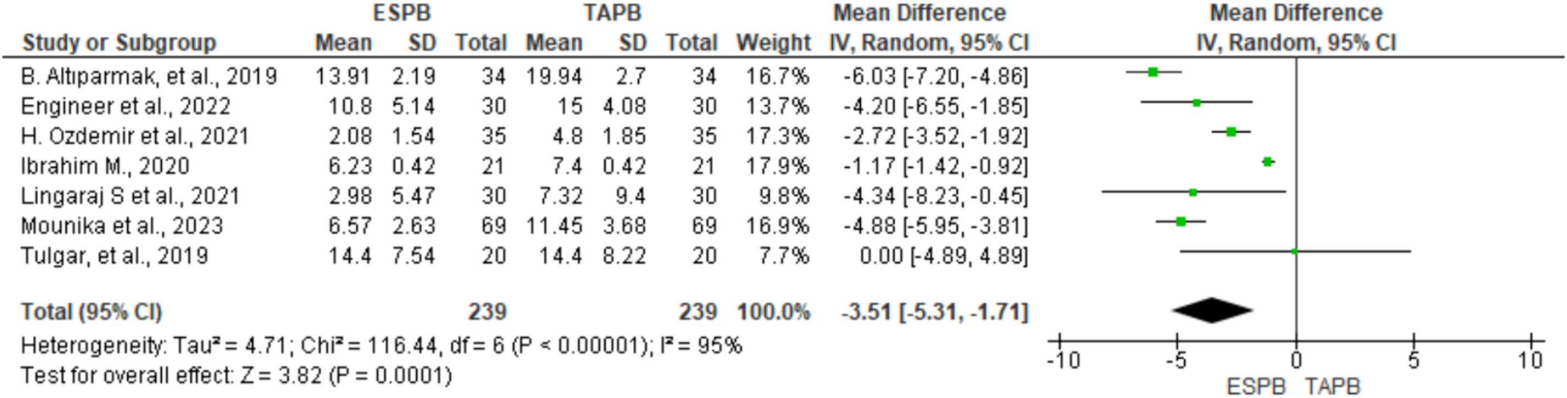
Forest plot diagram showing IV morphine equivalent consumption at 24 h postoperatively after LC.

Seven RCTs comprising 478 participants reported opioid consumption at 24 h postoperatively following LC. Four trials (18, 20, 21, 23) included IV tramadol, and others used fentanyl (22) and morphine (24, 25) to control postoperative pain. All opioids were converted to morphine equivalents (mg) to simplify the data analysis. Given the significant heterogeneity (Chi^2^ = 116.44, df = 6, I2 = 95%, *p* < 0.001), a random-effects model was used. The results of the meta-analysis indicated that the ESPB significantly lowered morphine equivalent consumption at 24 h postoperatively (MD = −3.51, 95% CI: −5.31 to −1.71; *p* < 0.001) than the TAPB group (Figure 5).

#### 3.4.3 First analgesia request time in minutes

**Figure 6:**
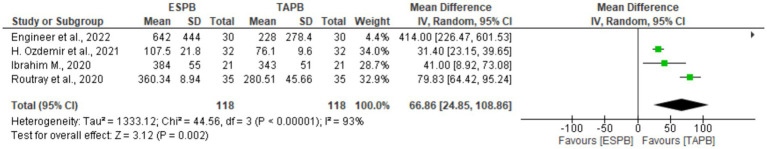
Forest plot diagram of first analgesia request time in minutes between groups after LC.

Four studies (18, 22, 24, 25) with 238 patients reported the first analgesia request time after LC. There was significant heterogeneity (Chi2 = 44.56, df = 3, I2 = 93%, p < 0.001); therefore, a random-effects model was used. The pooled results showed a significant difference between the two groups in the time to request first analgesia (MD = 66.86, 93% CI: 24.85 to 108.86; *p* = 0.002), as shown in Figure 6.

#### 3.4.4 Incidence of postoperative nausea and vomiting (PONV)

Five trials (19–21, 24, 25) provided information about the incidence of PONV at 24 h following LC. There was no significant heterogeneity (Chi^2^ = 7.13, df = 4, I^2^ = 44%, *p* = 0.13); however, a random-effects model was used. The pooled results revealed that there was no significant difference between the groups in the incidence of PONV (RR 0.70, 95% CI: 0.29 to 1.70, *p* = 0.43).

## 4 Discussion

**Figure 7:**
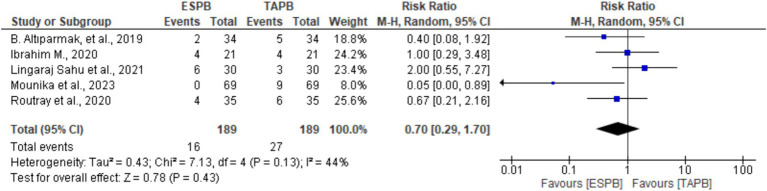
Forest plot diagram showing the incidence of postoperative nausea and vomiting after LC.

To the best of our knowledge, this is the first systematic review and meta-analysis that compares the efficacy of erector spinae plane block and transversus abdominis plane block for postoperative pain relief after laparoscopic cholecystectomy (see Figure 7).

According to our study, the ESP block was more effective in reducing pain severity scores at all measured time points after the surgery, both at rest and during movement, except for the 1st and 6th h of pain scores during movement. In addition, it resulted in lower morphine equivalent consumption during the first 24 h than the TAP block following LC. The ESP block also extended the time before the first request for additional pain relief compared to the TAP block. There were no significant differences in the incidence of postoperative nausea and vomiting. However, it is important to interpret the findings of this meta-analysis with caution, as the available data are of moderate quality and quantity.

The mean difference in pain scores, as measured on a 0–10 point analog scale, during rest and on movement consistently remained close to 0.5 cm at all postoperative times. Although this difference was statistically significant, it was not considered clinically relevant. It is worth mentioning that Bahreini et al. (30) have suggested that for a change in pain severity to be considered clinically significant, there should be a difference of more than 1.65 out of 11 on the NRS or 16.55 out of 100 on the VAS.

Consistent with our findings, a recent systematic review and meta-analysis (17) that included 10 randomized controlled trials (RCTs) with 570 patients who underwent various types of abdominal surgeries found that the ESP block significantly reduced pain scores at all-time points and opioid consumption during the first 24 h postoperatively compared to the TAP block; however, the observed difference was not clinically significant. Additionally, another recent Cochrane review (31) that included 64 studies found no clinically significant reduction in postoperative pain at rest 24 h after surgery in patients who received the ESP block. Another recent meta-analysis (32) compared different nerve blocks, including TAP, ESP, quadratus lumborum, paravertebral, and rectus sheath blocks, to provide effective postoperative analgesia for LC; however, our study is the first to compare ESP and TAP blocks for LC.

Although the ESP block has limited clinical significance in reducing pain scores, it does reduce opioid consumption, prolongs analgesia duration, and has minimal block-related adverse events (33). Our meta-analysis has shown that the ESP block can significantly decrease morphine cumulative consumption and enhance the first analgesia request time compared to the TAP block.

While a laparoscopic cholecystectomy (LC) is considered a minimally invasive procedure compared to an open cholecystectomy, it is still associated with significant postoperative pain that can increase readmission rates and hospital stays (4–6). Acute postoperative pain resulting from LC includes somatic, visceral, and referred pain (2, 3). Typically, patients experience the most intense pain within the first 24 h after surgery, with visceral pain being the primary type, followed by somatic pain (34). Additionally, patients who experience more intense visceral pain face a higher risk of developing chronic pain after undergoing LC (35).

In this context, the ESP block may prove to be more effective than the TAP block in treating somatic and visceral pain. The TAP block is limited to treating pain originating solely from somatic sensory, whereas the ESP block offers broader pain control (36). The mechanism of visceral analgesia achieved by ESPB is not yet fully understood, although limited evidence supports the theory that local anesthetics spread anteriorly into the paravertebral space, thereby blocking visceral sensory (37). Unlike the TAP block, the efficacy of the ESP block in blocking visceral pain pathways is subject to scrutiny and requires further research.

This meta-analysis has several limitations that should be noted. Like most systematic reviews, there is heterogeneity among the included studies. This heterogeneity is likely due to the various intraoperative and postoperative analgesic protocols used. Blocks were performed in different ways, either in awake states or before surgical incisions under anesthesia, and we combined all these approaches. Although all the trials included in this study were of high quality, they had small sample sizes, which may impact the level of evidence presented. Additionally, a variety of local anesthetic drugs, volumes, and dosages, with or without adjuvants, were used across the studies, which could have influenced the results. Finally, due to a lack of access, we used the risk of bias tool 1 (ROB 1) instead of the risk of bias tool 2 (ROB 2).

Despite these limitations, this meta-analysis is the first to pool results from recent randomized controlled trials published within the last 5 years, comparing the efficacy of erector spinae plane and transversus abdominis plane blocks for pain control following laparoscopic cholecystectomy.

## 5 Conclusion

The ESP block effectively reduces postoperative pain scores at rest and during movement. However, it has not yet reached a level of clinical significance. Furthermore, the use of the ESP block leads to a significant reduction in morphine equivalent consumption and improves the time at which patients first request analgesia after laparoscopic cholecystectomy. We suggest clinicians consider using an ultrasound-guided ESP block as an effective technique for postoperative pain relief in patients undergoing LC.

## Data availability statement

The original contributions presented in the study are included in the article/Supplementary material, further inquiries can be directed to the corresponding author.

## Author contributions

DZ: Conceptualization, Data curation, Formal analysis, Funding acquisition, Investigation, Methodology, Project administration, Resources, Software, Supervision, Validation, Visualization, Writing – original draft, Writing – review & editing. TTa: Conceptualization, Data curation, Formal analysis, Funding acquisition, Investigation, Methodology, Project administration, Resources, Software, Supervision, Validation, Visualization, Writing – original draft, Writing – review & editing. SE: Conceptualization, Data curation, Formal analysis, Funding acquisition, Investigation, Methodology, Project administration, Resources, Software, Supervision, Validation, Writing – original draft, Writing – review & editing. TTi: Conceptualization, Data curation, Formal analysis, Funding acquisition, Investigation, Methodology, Project administration, Resources, Software, Supervision, Validation, Visualization, Writing – original draft, Writing – review & editing.
