# Supplementary material for: Analgesic efficacy of erector spinae plane block versus transversus abdominis plane block for laparoscopic cholecystectomy: a systematic review and meta-analysis of randomized controlled trial
Source: Front Med (Lausanne). 2024 Jul 29;11:1399253. doi: 10.3389/fmed.2024.1399253 (PMC11317285; doi:10.3389/fmed.2024.1399253)
Supplement: Supplementary file 1 [file Data_Sheet_1.docx]

**Supplementary file 1**

**PubMed**

"Erector spinae plane block*" OR "transversus abdominis plane block*" AND "' laparoscopic cholecystectomy*'"

[**Embase**](https://go.openathens.net/redirector/www.monash.edu?url=http://ovidsp.ovid.com/ovidweb.cgi?T=JS%26MODE=ovid%26PAGE=main%26NEWS=n%26DBC=y%26D=EMCZD)

"Erector spinae plane block*".mp. AND transversus abdominis plane block/ or "transversus abdominis plane block*".mp. AND cholecystectomy/ or laparoscopic cholecystectomy/

[**Scopus**](https://go.openathens.net/redirector/www.monash.edu?url=https://www.scopus.com)

TITLE-ABS-KEY (“Erector spinae plane block*" OR "transversus abdominis plane block*" AND "'laparoscopic cholecystectomy*'" OR "'cholecystectom*'" )

[**CINAHL**](https://search.ebscohost.com/login.aspx?profile=ehost&custid=s8849760&authtype=shib&defaultdb=ccm)

"Erector spinae plane block*".mp. AND "transversus abdominis plane block*".mp. AND "'laparoscopic cholecystectomy*'".mp. OR "'cholecystectom*'".mp
